# Analysis of the Interaction between Lake and Groundwater Based on Water–Salt Balance Method and Stable Isotopic Characteristics

**DOI:** 10.3390/ijerph191912202

**Published:** 2022-09-26

**Authors:** Changming Cao, Na Li, Weifeng Yue, Lijun Wu, Xinyi Cao, Yuanzheng Zhai

**Affiliations:** 1 College of Water Sciences, Beijing Normal University, Beijing 100875, China; 2 China Irrigation and Drainage Development Center, Beijing 100054, China

**Keywords:** water–salt balance method, stable isotope, Bayesian mixed model, surface water–groundwater interaction, water ecology

## Abstract

To better protect lacustrine ecologies and understand the evolutionary process of lake environments, it is critical to study the interacting mechanisms between lakes and the surrounding groundwater. The Wuliangsu Lake watershed is the largest wetland in the Yellow River basin and is the discharge area of the Hetao Irrigation District (HID), which is one of the three largest agricultural production areas in China. Due to the influence of human activities, the discharge water from the HID has led to the deterioration of the Wuliangsu Lake ecology and the degradation of the lake environment. Based on long-term observation data and water sampling data collected in 2021, a water–salt equilibrium model was used to analyze the recharge rate of groundwater to the lake. The contribution rate of groundwater to lake recharge in the study area was calculated with a Bayesian mixing model by combining D and ^18^O stable isotope data. Furthermore, the environmental evolutionary process of the lake was also analyzed using the collected water quality data. The results show that channel drainage was the main source of recharge to Wuliangsu Lake, accounting for more than 75%, while groundwater contributed less than 5% of lake recharge. After implementing the ecological water supplement plan, the concentration of various ions in the lake decreased, the concentration of the total dissolved solids (TDS) in the lake decreased from 1.7 g/L in 2016 to 1.28 g/L in 2021, and the ecological environment was improved. The contribution of groundwater to lake recharge was quantitatively analyzed. The results of this study can facilitate the development of vital strategies for preventing the further deterioration of lake water quality and for protecting wetland ecologies.

## 1. Introduction

Rivers, lakes, wetlands and other surface water are connected with groundwater sources, and the interaction between groundwater and surface water is an important consideration in wetland ecology and environmental protection [1,2,3,4]. Since joining the Ramsar Convention, the Chinese government has attached great importance to the protection of natural wetlands and implemented the National Wetland Conservation Program (NWCP) [5]. This further emphasizes the importance of wetland protection and puts forward higher requirements for the interaction mechanism between groundwater and surface water. Therefore, it is crucial to study the interactive mechanisms between lakes and the surrounding groundwater to protect lake environments and prevent the further deterioration of lake ecology.

Though stable isotope analysis has been increasingly applied in a wide range of fields in ecology and biology, the study of the hydrologic cycle represents one of the earliest applications of stable isotopes [6]. Globally distributed hydrogen and oxygen isotope data indicate that ecohydrological separation is widespread across different biomes [7]. In recent years, attention has been given to the role of stable isotopes in the water cycle of wetland systems, which has influenced ecological and hydrological theories [8]. Among them, the stable isotopes D and ^18^O are helpful for the quantitative assessment of groundwater recharge, flow. and other processes and are the ideal isotopes for studying water bodies. Based on the composition characteristics of D and ^18^O isotopes in different water bodies, the source, migration, mixing, and other hydrologic processes can be analyzed [9]. Natural isotope tracing can also be used to identify and quantify various water catchment input pathways, including sources of various water types, based on calculations of lacustrine water balance, precipitation, and river inputs [10,11]. Stable isotopes are increasingly used as tracers in environmental studies. One application is to use isotope ratios to quantitatively determine the proportional contribution of several sources to the mixed water mass, such as the proportions of various sources of pollution in a runoff stream [6]. In recent years, MixSIAR, an R package that can create and run Bayesian mixing models to analyze stable isotopes, has unified the existing set of mixing model parameterizations into a customizable tool that can meet the needs of most studying mixing systems [12]. The results of Mix SIAR can be used to evaluate the contribution of advection, transpiration and evaporation to the basin’s water cycle [13] and to quantify the relative contribution of flow in the unsaturated groundwater zone [14]. The development of a lake water and salt mixing model is helpful to analyze the contributions of various water bodies [15] and to understand the relationship between water and salt migration in groundwater and surface water systems, thereby identifying the process of water quality changes Then, specific protective measures can be proposed, thus facilitating rational use of surface water and groundwater [16].

Previous studies were more focused on the application of a single method such as a simulation model, water balance model or isotope from the perspective of water quantity. In this study, Wuliangsu Lake, located in the western Inner Mongolia region of China, was selected as the study area, and a water–salt equilibrium model combined with the isotope method was applied to analyze the interaction between surface and ground water in view of water quantity and quality. Then, the rate of groundwater contribution to the Wuliangsu Lake water supply was calculated by using stable isotopes and a water–salt equilibrium model, and the ecological evolution of the Wuliangsu Basin was also analyzed by using the water–salt equilibrium and water chemical characteristics. This study provides an important theory for preventing the further deterioration of lake water quality and wetland ecological protection.

## 2. Materials and Methods

### 2.1. Study Area

Wuliangsu Lake (40°47′–41°03′ N, 108°43′–108°57′ E), located in western Inner Mongolia, China, is one of the largest freshwater lakes in northern China and has the largest wetland area in the Yellow River Basin with an area of 370 km^2^ [17]. The multiyear average elevation of the lake surface is 1018.8 m, with an average capacity of 3.0 × 10^8^ m^3^. The mean depth of the lake is approximately 1.0 m. The study area has a continental climate in the middle temperate zone, with an annual average rainfall of approximately 200 mm and evaporation of more than 2000 mm. The annual average temperature is 6.8~9.3 °C, and the annual average wind speed is 2.20 m/s. The long-term minimum and maximum air temperatures are −20 °C in winter and 35 °C in summer, respectively. 

The Hetao Irrigation District (HID), which is located in the upstream of Wuliangsu Lake, is the largest agricultural production area along the Yellow River basin with an area of 1.12 × 10^6^ ha [18]. The HID has carried out long-term and large-scale development of water resources [19]. Due to the influence of human activities, a large number of ditches have been developed for irrigation and drainage [20], and agricultural wastewater is drained into Wuliangsu Lake. Wuliangsu Lake receives water (approximately 5.5 × 10^6^ m^3^/a) from the HID through the drainage channels and discharges approximately 1.9 × 10^6^ m^3^/a to the Yellow River through the main channel, thereby leading to the deterioration of the lake environment [21]. Therefore, the ecological environment of Wuliangsu Lake is experiencing serious degradation.

### 2.2. Sampling and Measurements

#### 2.2.1. Sampling and Conservation

**Figure 1:**
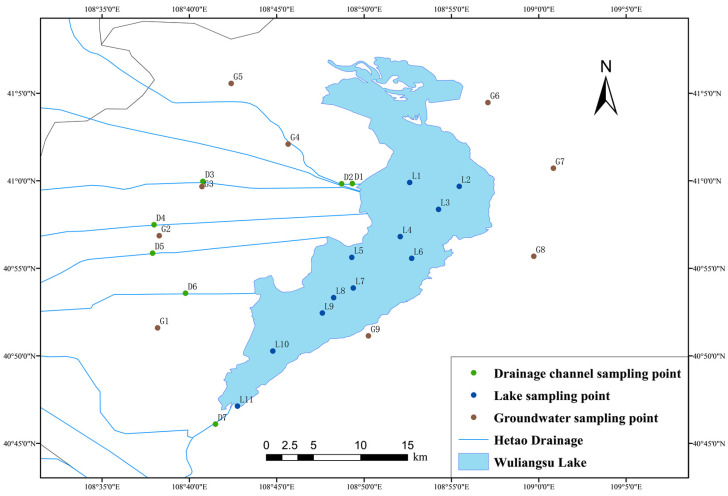
Distribution of the sampling sites in the study area.

Water samples were collected in April and October 2021. Samples were collected from the lake, drainage channels, and groundwater, of which 10 samples were from lake water, 9 samples were from groundwater, and 8 samples were from the drainage channels, as shown in Figure 1. The lake water sampling site covered the entire lake, groundwater sampling was conducted through the monitoring wells, and drainage water samples were located near the entrance of the lake. All water samples were sealed in polyvinyl chloride bottles and transported to the laboratory as soon as possible, where they were then filtered through a 0.45 μm membrane and stored in a refrigerator at 4 °C.

#### 2.2.2. Measurements and Analysis

The total phosphorus content in the water samples was determined using a plasma emission spectrometer (ICP-OES, Spectro Arcos, BIC, DEU). Ion chromatography (ICS-2100, Sunnyvale, CA, USA) was used to measure the ions in the water samples, including four cations (K^+^, Na^+^, Ca^2+^, and Mg^2+^) and three anions (Cl^−^, NO_3_^−^, and SO_4_^2−^).

The stable isotope ratios ^2^H (D) and ^18^O of water molecules in the samples were measured by use of a liquid water isotope analyzer (Picarro L2140-i, Santa Clara, CA, USA). Each sample was analyzed six times. To minimize the memory effect, the results of the first two analyses were discarded. The measured results were part per thousand deviations from the Vienna Standard Mean Ocean Water, and the measurement accuracies for δD and δ^18^O were 0.50‰ and 0.15‰, respectively.

### 2.3. Study Method

The study objectives were accomplished by the use of multiple methods. The water balance method is the most common method used to estimate groundwater recharge. This method is an indirect approach that uses the water equilibrium equation. However, the quantity of groundwater recharge was unknown, though the other equilibrium concentrations needed to calculate the groundwater recharge were known. 

The water recharge source of the lake is mainly from irrigation and drainage in the HID. In addition, atmospheric precipitation and underground runoff also contribute to lake recharge. The lake primarily loses water via evaporation, followed by drainage and leakage. The sluice at the southern end of the lake drains the water into the main drainage channel and then discharges it into the Yellow River. Therefore, in the water balance equation for the study area, the recharge water includes channel drainage, rainfall, and groundwater lateral inflow, while discharge water includes drainage into the Yellow River, evapotranspiration, and groundwater leakage. Therefore, the amount of groundwater recharge to the lake is:

(1) $$G_{i}=\Delta w-\left(P+R\right)+\left(ET+O+G_{o}\right)$$

where *G_i_* is the groundwater lateral inflow, Δ*w* is the annual water storage change in Wuliangsu Lake, *P* is precipitation, *R* is channel drainage; *ET* is evapotranspiration, *O* is the outflow from the lake through the main channel, and *G_o_* is groundwater leakage.

The precipitation, temperature, wind speed, solar radiation, and relative humidity data in the study area were obtained from the weather station in Urad Front Banner. The calculation of evaporation is based on the Dalton theorem and combined with meteorological factors to calculate water surface evaporation:

(2) $$E_{w}=0.22\times (e_{s}-e_{a})\times \sqrt{1+0.32u^{2}}$$

where *E_w_* is the water surface evaporation in mm during the non-ice period (from April to October); *e_s_* is the saturated vapor pressure at the water surface temperature, hPa; *e_a_* is the actual water vapor pressure, hPa; and *u* is the surface wind speed, m/s.

The empirical formula of ice period evaporation obtained by Bayangaole Evaporation Experimental Station is as follows [22]:

(3) $$E_{i}=-36.82\times \left(T^{2}\times W^{2}/U^{2}\right)+56.75$$

where *E_i_* is the monthly evaporation during the ice period (from November to March), mm; *T* is the monthly average temperature, °C; *W* is the monthly average wind speed, m/s; and *U* is the monthly average relative humidity, %.

The calculation of evapotranspiration in Wuliangsu Lake can be divided into two parts: the water surface area and reed area. The evapotranspiration in the water surface area can be calculated in the ice period and non-ice period according to the above formula. The calculation of the reed area was also divided into two seasons: one was the rapid growth season, and the other was the season where reeds ceased to grow. The rapid growing season corresponded to the lake’s non-ice period, which was from April to October. According to the study of the reed growth season in the Zhalong Wetland [23,24], the proportion coefficient of evapotranspiration compared with the water surface area is 1.1. The season of reed growth cessation corresponded to the ice period of the lake, and there was almost no evapotranspiration during this interval. Hence, the evapotranspiration of the reed area was based on the evapotranspiration of the water surface area in the ice period.

Through sampling and analyzing the stable isotope characteristics of different water types (channel drainage, groundwater, meteoric precipitation, and lake water) in the Wuliangsu Lake Basin, the contribution rate of different water bodies to lake water recharge was calculated according to the mass conservation principle of the stable isotope.

(4) $$\delta_{M}=f_{1}\left(\delta_{1}+\gamma_{1}\right)+f_{2}\left(\delta_{2}+\gamma_{i}\right)+\ldots f_{n}\left(\delta_{n}+\gamma_{i}\right)$$

where *δ_M_* is the stable isotope value of lake water, *f_i_* is the contribution proportion of different recharge water bodies to the mixture, *δ_i_* is the stable isotope value of different recharge water bodies, and *γ_i_* is the fractionation value between the stable isotope values of different recharge water bodies and lake water.

Solute transport and hydrogeochemical processes occur continuously in the water cycle in the basin, and the value of the hydrogeochemical index is the comprehensive result of various hydrogeochemical processes. The total dissolved solids (TDS) of each water body are usually used to calculate the salt migration process in the basin, so as to calculate the contribution rate of groundwater seepage to lake recharge. The change in salt content of Wuliangsu Lake (Δ*S_L_*) is as follows:

(5) $$\Delta S_{L}=S_{Y}+S_{C}+S_{G}-S_{D}$$

where *S_Y_* is the salt content of ecological water replenishment; *S_C_* is the salt content of the drainage channel; *S_G_* is the salt content of groundwater; and *S_D_* is the amount of salt discharged from the lake through the channels.

From δ^18^O and δD stable isotope information, the MixSIAR model, based on Bayesian theory, was used to analyze the contribution rate of different water bodies to the Wuliangsu Lake recharge. The stable isotope characteristics of the lake were used as mixing terms in the analysis model, and the recharge terms included groundwater, channel drainage, and atmospheric precipitation. Atmospheric precipitation isotopes were derived from the Baotou monitoring site as reported by the Global Precipitation Isotope Network (GNIP), a monitoring network established by the International Atomic Energy Agency (IAEA) and the World Meteorological Organization (WMO). Stable isotope values of channel drainage, groundwater, and lake water were obtained from sampling data. The input data of the MixSIAR model include source data (the δ^18^O and δD stable isotope values of atmospheric precipitation, channel drainage, and groundwater) and mixture data (the δ^18^O and δD stable isotope values of the lake). According to the calculation results, the corresponding median contribution ratio of each recharge water body is regarded as the contribution rate of this water body to the lake recharge.

## 3. Results and Discussion

### 3.1. Water and Salt Balance Analysis

#### 3.1.1. Analysis of Equilibrium Elements of Water and Salt

1. Precipitation and evapotranspiration

**Figure 2:**
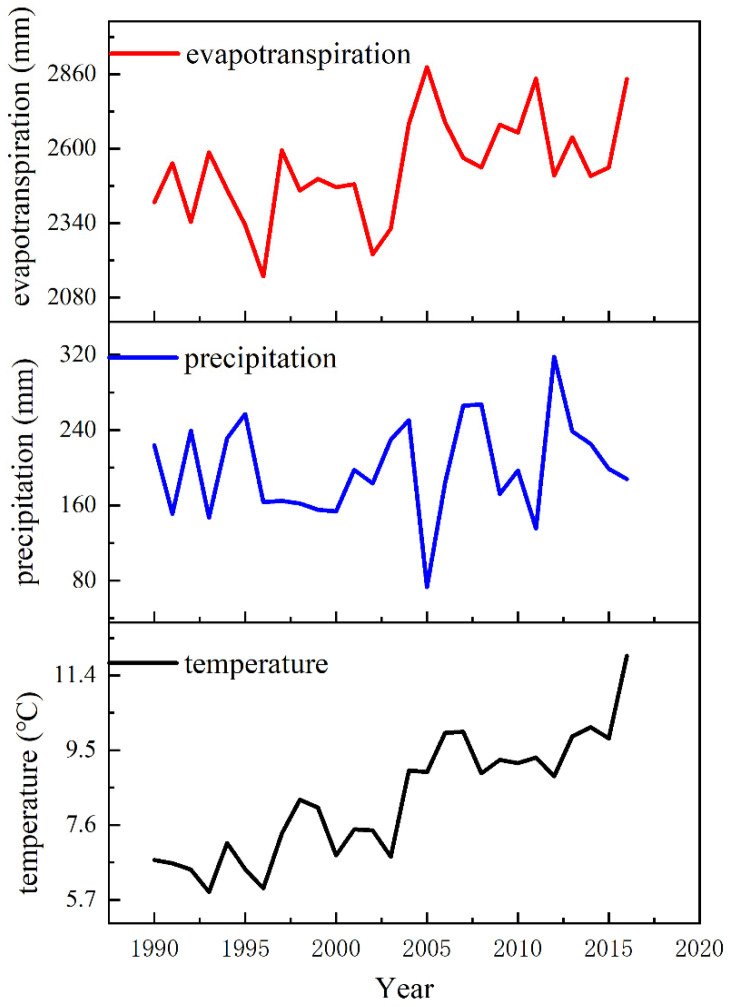
Analysis of temperature, rainfall and evapotranspiration in the study area.

The changes in temperature, precipitation, and evapotranspiration in the Wuliangsu Lake region were based on the monitoring data from the Urad Front Banner meteorological station between 1990 and 2016 and based on the principle of evapotranspiration over the water surface of Wuliangsu Lake. These results are shown in Figure 2.

Precipitation is an important supplemental water source of the lake in this region, and the amount of precipitation is closely related to the change in the lake water level [25,26]. There is a small precipitation range over the lake basin. Compared with evapotranspiration, precipitation in the basin remains at a low level year-round. According to the statistical data of precipitation from 1990 to 2016, the average annual precipitation in the lake basin was 199.01 mm. By analyzing the evapotranspiration curve, it can be seen that the annual evapotranspiration volume of the lake generally showed an upwards trend at a rate of 57.90 mm/a and five-year cycle. The interannual variation of lake basin temperature was similar to that of evapotranspiration, showing an upwards trend with a rate of 0.17 °C/a. In terms of water volume, the average annual precipitation was much lower than the amount of moisture lost to evapotranspiration, with a difference of more than 2000 mm. Looking at these trends specifically, concurrent with the influence of human activities and global climate change, evapotranspiration and temperature increased each year, while precipitation had the opposite change, showing a slight downwards trend over the analysis period.

2. Channel recharge and discharge

**Figure 3:**
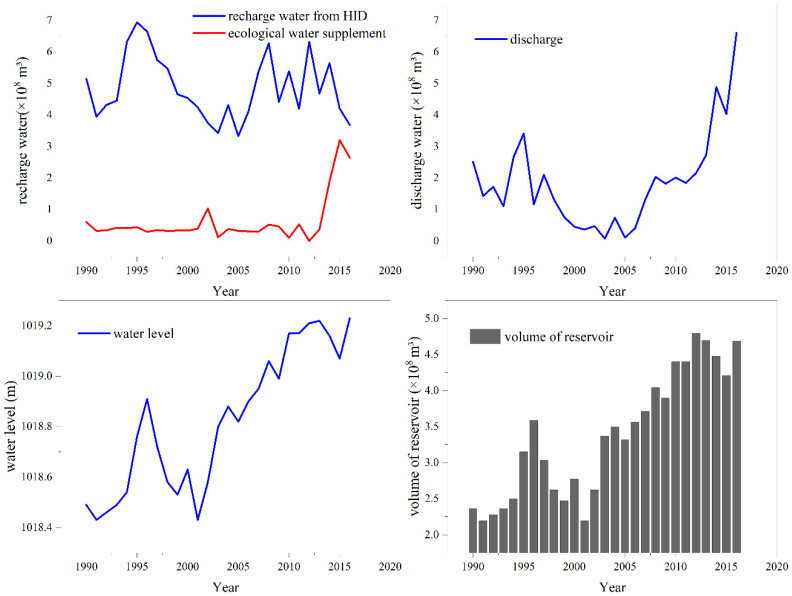
Analysis of water supply and displacement of the lake through channels and schematic diagram of water level storage capacity change.

Wuliangsu Lake was selected to systematically analyze lake recharge and discharge. Under the influence of human activities, lake water recharge sources mainly included farmland drainage from the HID and ecological water supplements, while the water lost by the lake was mainly due to drainage into the Yellow River. Figure 3 shows the long-term changes in water supply and loss in the lake under the influence of human activities while also showing the changes in storage capacity and water level in the lake from 1990 to 2016.

As seen from Figure 3, from 1990 to 2016 the average annual recharge water volume was 4.87 × 10^8^ m³; however, the volume of recharge water from the HID was highly variable with no obvious upwards or downwards trend. The ecological water supplement refers to the water directly replenished by the Yellow River to Wuliangsu Lake. Before 2013, the ecological water supplement was quite low and then increased significantly to approximately 0.88 × 10^8^ m³/a. The water discharge into the Yellow River initially showed a decreasing trend but then increased from 1990 to 2016. The decrease was mainly due to the increasing demand for agricultural irrigation water in the HID. The increase was mainly controlled by two aspects: one was the need to dilute the salt content of the lake, and the other was the increase in recharge water from the channel. Lake volume increased in three stages from 2005 to 2016. The first stage was from 2005 to 2008; during this period, the amount of water discharge increased rapidly. The second stage was from 2009 to 2012, the water discharge varied little, and the water recharge in the corresponding irrigated area showed no obvious upwards trend. The third stage was from 2013 to 2016, when the amount of water discharge rose rapidly, though the main influencing factor was the large increase in ecological water supplementation. The water level increased slowly with time at a rate of 0.03 m/a. The reservoir volume of the lake showed a variable upwards trend, which was greatly affected by the ecological water supplement and drainage.

#### 3.1.2. Equilibrium Results and Contribution Rate Analysis

According to a previous study [27], the lateral discharge of the lake to groundwater through the underground medium was, on average, 0.66 × 10^8^ m³ for many years. During the period from 1990 to 2016, the change in lake storage capacity was 0.09 × 10^8^ m³.

Based on the value of each balance item, the groundwater recharge to Wuliangsu Lake can be calculated with the water balance method, as shown in Table 1. Table 1 shows that the multiyear average amount of groundwater recharge to the lake was 0.25 × 10^8^ m³, and the contribution rate of groundwater to the total recharge of Wuliangsu Lake accounted for only 3.96%.

**Figure 4:**
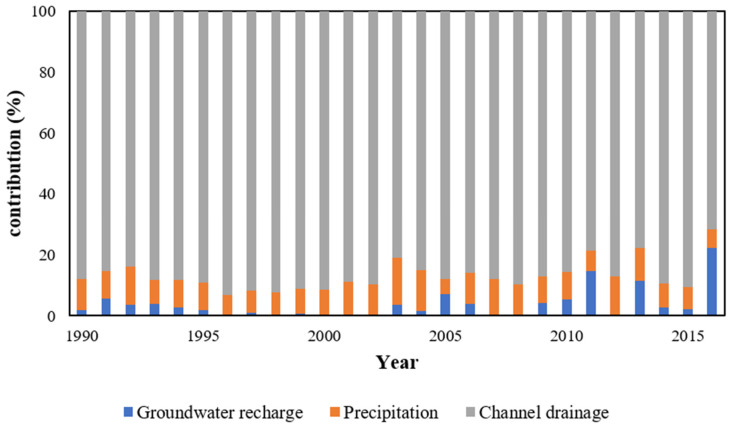
Changes in recharge to Wuliangsu Lake from 1990 to 2016.

Based on the analysis of various recharge elements of the lake over the years, as shown in Figure 4, it can be seen that channel drainage, which contributed more than 80% of the total recharge to the lake, played a dominant role in various inflows into the lake. Following channel discharge, precipitation accounted for 10% to 20% of the contribution. However, the multiyear average contribution of groundwater to the lake was very small (only 3.96%); that is, there was no strong interaction between groundwater and the lake. Furthermore, the annual variation in the contribution of groundwater appeared very unstable, which may be closely related to lake water storage and climate change.

**Figure 5:**
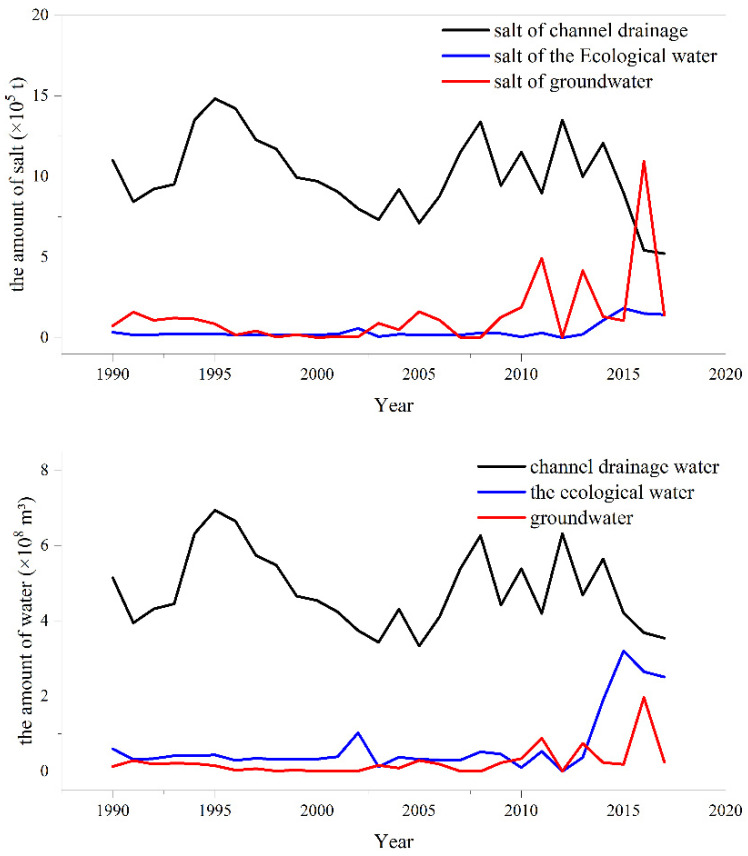
The water and salt evolution of Wuliangsu Lake.

Figure 5 shows the changes in lake salt content over time as it relates to lake recharge variability by recharge type. The TDS value in groundwater was relatively high and stable; however, the proportion of groundwater to overall water recharge was very low. Therefore, the salt carried by groundwater also had a small proportion in the overall salt recharge of the lake. From 1990 to 2016, the average annual salt recharge from groundwater was 3.92 × 10^4^ t, accounting for approximately 3.23%. Farmland drainage was one of the most important sources of water in the lake, and it was the main source of lake salt due to its high salinity, accounting for approximately 85.1%. In addition, with the decrease in farmland water discharge and TDS values in recent years, the salt supply of Wuliangsu Lake from this equilibrium term also decreased each year. Consistent with a recent decrease in salinity, the ecological recharge water (the TDS value was approximately 0.676 g/L) has gradually increased in recent years, and has played an important role in the ecological treatment of the lake.

**Figure 6:**
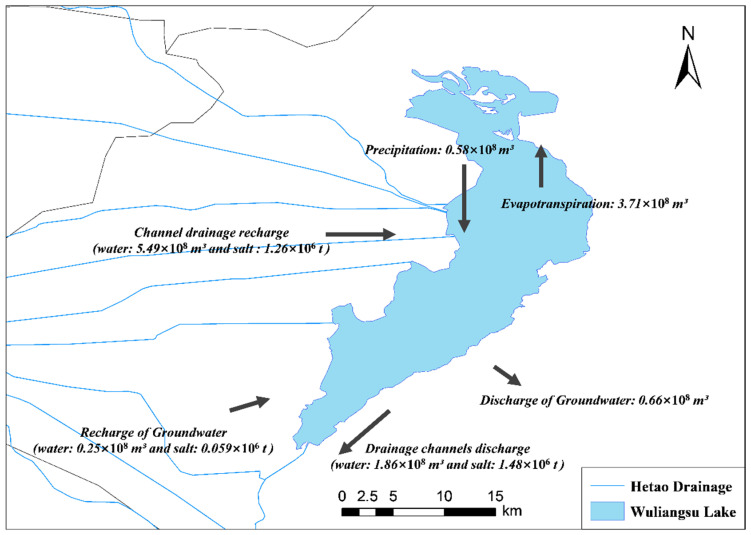
Diagram of water and salt equalization in Wuliangsu Lake.

Figure 6 integrates the process of water and salt transport in the lake system and plots the water–salt equilibrium model of the lake system from 1990 to 2016. As seen from Figure 6, because the groundwater supply to the lake was minimal, there was only a weak connection between groundwater and surface water, and the effect of groundwater on ecological conditions was limited. Between 1990 and 2016, the average annual salt supply of the lake was 1.32 × 10^6^ t, and the average annual salt discharge was 1.48 × 10^6^ t. Wuliangsu Lake underwent a period of desalinization (salt reduction) due to the implementation of the ecological water management program. The data show that the TDS value of the lake was 1.7 g/L from 2011 to 2016 [28], and according to recent sampling data, the TDS value of the lake decreased further to 1.28 g/L from 2016 to 2021, a decrease of approximately 25%. Therefore, the implementation of the ecological water supplement plan effectively helped to desalinate Wuliangsu Lake and promoted improved ecological and environment management of the basin.

### 3.2. Analysis of Stable Isotope Characterization

#### 3.2.1. Stable Isotope Characteristics of Different Water Sources

**Figure 7:**
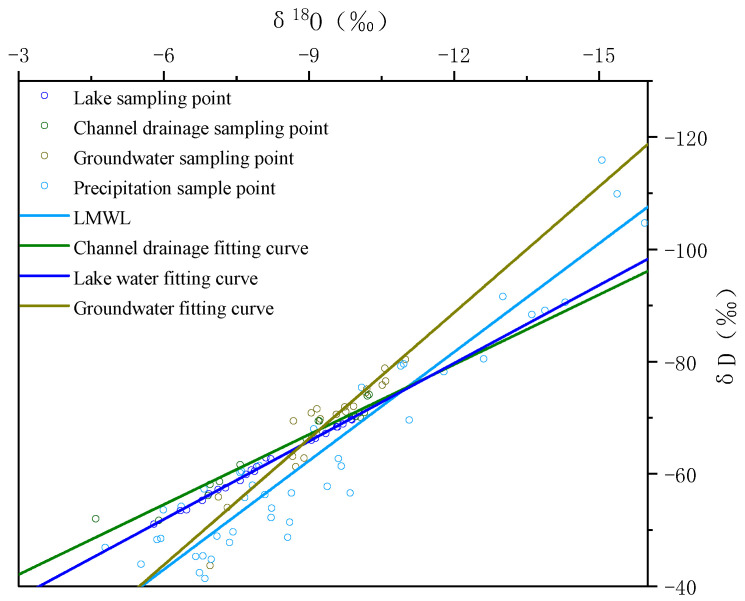
Relationships among δ^18^O and δD of channel water, lake, groundwater, and local precipitation in the Wuliangsu Lake basin.

Figure 7 depicts the hydrogen and oxygen stable isotope relationship curves of different water bodies as derived from the water sample tests in April (Table S1) and October (Table S2) 2021 and the observed stable isotope characteristics of atmospheric precipitation in Baotou. The δD values of groundwater, channel drainage, and precipitation were −68.47‰, −64.95‰, and −57.92‰, respectively. Given that the δD of lake water was −61.82‰, the hydrogen isotope value of groundwater was more negative than that of lake water. According to the hydrogen isotope values, the recharge contribution relationship was preliminarily determined as follows: channel drainage, precipitation, and groundwater recharge contribution decreased successively.

Comparisons of the variation in water samples collected in different seasons (April and October) shows that groundwater had the least seasonal variability at only 0.8%. The range of variability for channel drainage water was 3.49%, which was mainly affected by human activities. The lake water had the largest range, which was 12.26% between the autumn and spring. There were two main reasons for this: on the one hand, the lake water was more affected by the natural environment, and the stable isotope characteristics in the lake water were easily affected by the change in stable isotopes as driven by precipitation. On the other hand, due to intense summer evaporation, the stable isotope values of hydrogen and oxygen in October were more negative than those in April.

The global precipitation isotope observation network GNIP established by IAEA and WMO contains precipitation δD and δ^18^O data from 27 monitoring points in China. According to the geographical location, the monitoring site in Baotou is the closest to the actual study area. The mean values of δD and δ^18^O of precipitation in the Baotou area are −57.92‰ and −8.31‰, respectively. The approximate local precipitation line equation (LMWL) in the study area is shown in Figure 7, and the fitting curve formula is as follows:

(6) $$\delta D=6.45\delta^{18}O-4.26$$

Figure 7 shows that the δD-δ^18^O values of lake water and channel drainage were different from those of groundwater. The fitting curve of lake and channel drainage water was closer to the local meteoric water line (LMWL) but showed a drift compared with the global meteoric water line (GMWL). The δD-δ^18^O fitting curve of lake water and channel drainage was gentler than that of groundwater. This indicates that the stable isotope characteristics of groundwater, lake water, and channel drainage differed greatly, and the recharge effect of groundwater was not obvious in the interaction relationship of the lake and groundwater.

#### 3.2.2. Stable Isotope Ratios for Water Exchange

**Figure 8:**
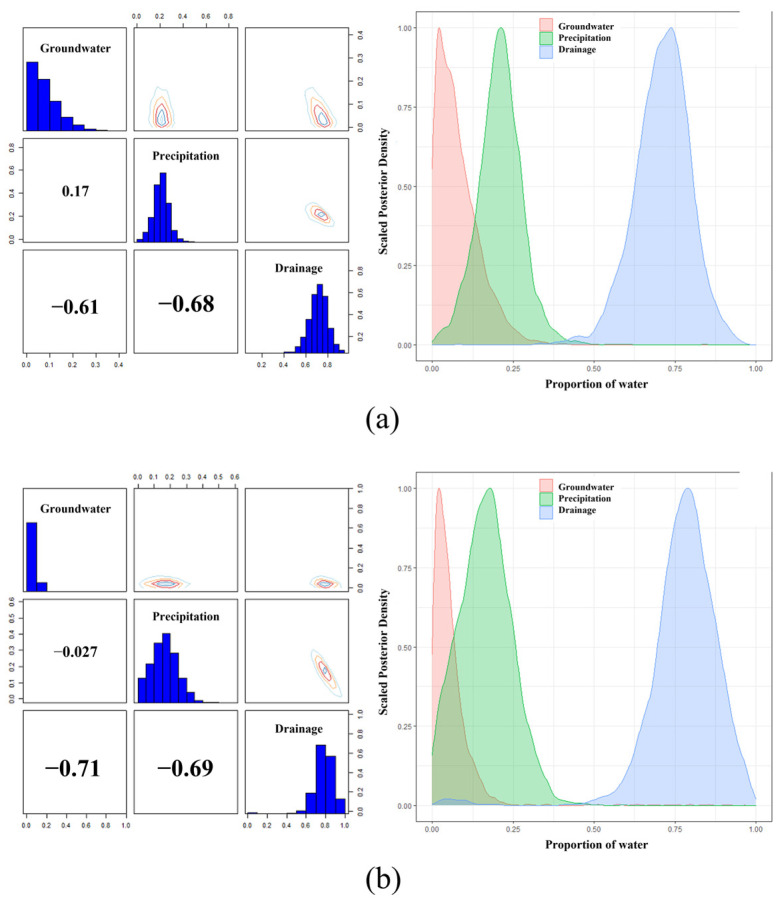
Contribution of the recharge of channel drainage, groundwater, and local precipitation to Wuliangsu Lake (**a**) in April 2021 and (**b**) in October 2021.

The Bayesian-based MixSIAR model was used to analyse the contribution rate of different water bodies. As shown in Figure 8, for April 2021, the left figure shows the correlation of contributions of different recharge sources to lake recharge. According to the results, channel drainage had a high correlation with precipitation and groundwater, while the correlation between groundwater and precipitation was low. Therefore, it can be inferred that channel drainage was related to the other two items. On the other hand, the contribution rates of groundwater, atmospheric precipitation, and channel drainage to lake recharge were 4.9%, 21.0%, and 74.1%, respectively (Table S3). In October 2021, groundwater, atmospheric precipitation, and channel drainage contributed 4.2%, 18.1%, and 77.7% to the recharge of the lake, respectively (Table S4). Therefore, channel drainage was the main recharge source of Wuliangsu Lake, and the groundwater recharge contribution was less than 5% according to the results of the stable isotope analysis.

Based on the water–salt balance model, the annual average contribution rate of groundwater to lake recharge was 3.96%. In addition, comprehensive analysis of the contribution rate of different water bodies to lake recharge with stable isotope method showed that the average contribution rate of groundwater, precipitation, and channel drainage to lake recharge were 4.55%, 19.55%, and 75.9%, respectively. This result was similar enough to that of the water–salt equilibrium analysis. Through the mutual verification of the two analytical methods, the conclusion in this study was sufficient to show the weak correlation of groundwater recharge to the lake. Therefore, more attention must be paid to the impact of channel drainage when considering ecological conservation and water resources management of the Wuliangsu Lake.

### 3.3. Hydrochemical Characteristics

#### 3.3.1. Seasonal Variation in Ecological Indicators

According to the statistical results of the main ions in each water body (Tables S5 and S6), the pH value of the lake water was 8.3–8.9, which is weakly alkaline. The concentration of Na^+^ was the highest among the cations, and the concentrations of Cl^−^ and HCO_3_^−^ were the highest among the anions. TDS ranged from 1000 to 3000 mg/L, with an average of 1853.90 mg/L. The pH value of groundwater was 7.5–8.5, and Na^+^ was the most abundant cation and was more abundant in the groundwater than in Wuliangsu Lake. The concentrations of Cl^−^ and HCO_3_^−^ in anions were also higher in the groundwater, and a large amount of nitrate was detected, with a maximum concentration of up to 224.22 mg/L. The mean concentration of TDS in groundwater was 5568 mg/L. The pH value of the drainage channel was 8.2–8.7, and it also contained high concentrations of Na^+^, Cl^−^, and HCO_3_^−^. The mean TDS concentration was 3492 mg/L, which was related to irrigation in the HID.

**Figure 9:**
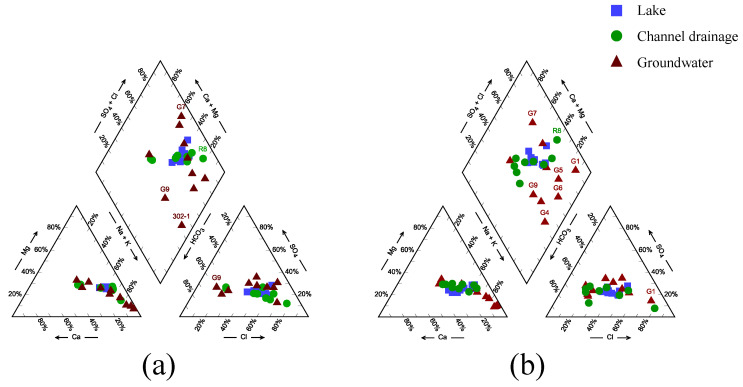
Piper diagram of the hydrochemical types in the three water bodies (**a**) in April 2021 and (**b**) in October 2021.

A Piper diagram (Figure 9) was drawn based on the hydrochemical characteristics of each water body. In spring (April), the anions in channel drainage and lake water were dominated by Cl^−^, while the cations were dominated by Na^+^, and the hydrochemical type was mainly Na-Cl. In autumn (October), the HCO_3_^−^ concentration in channel drainage and lake water increased significantly, and the water chemical type also changed from the Na-Cl type to the Na-HCO_3_ type, but the concentration of Cl^−^ was still above 35%.

In addition, by comparing the hydrochemical characteristics of different water bodies, there were significant differences in the hydrochemical types of lake water, drainage channel water, and groundwater. The hydrochemical types of lake water and drainage channel water were relatively close, and the main hydrochemical types were Na-Cl and Na-HCO_3_. The cations in groundwater were mainly Na^+^ and Ca^2+^, the concentration of Na^+^ ranged from 40% to 90%, and that of Ca^2+^ ranged from 5% to 60%. The anions were mainly controlled by Cl^−^ and HCO_3_^−^, of which the Cl^−^ concentration varied between 20% and 80%, while HCO_3_^−^ mainly ranged from 35% to 75%. The results showed that there was a substantial difference between groundwater and channel water, as well as lake water. This indicates that there was only a weak connection between groundwater and lake water in terms of the hydrochemical characteristics. 

**Figure 10:**
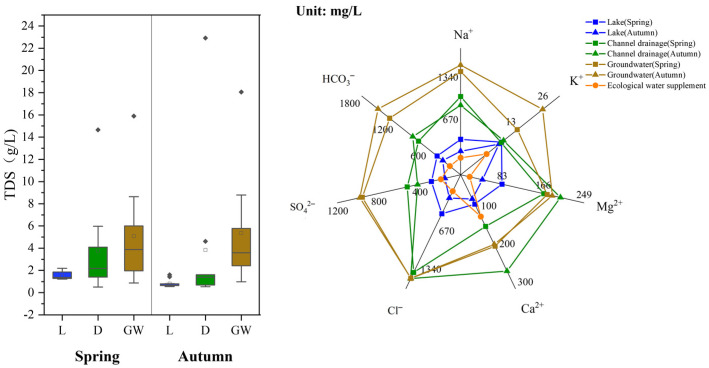
Changes in hydrochemical characteristics of the three water bodies in different seasons in 2021.

Based on the sampling results in April and October, Figure 10 shows the range of TDS values and changes in major ions in lake water, groundwater and channel drainage during different seasons during 2021. Compared to different water types, the average TDS of lake water in spring and autumn was 1.51 and 0.81 g/L, respectively, which was significantly lower than the TDS of channel drainage and groundwater. According to previous research results from 2019 [29], the average TDS of the lake water was between 1.3 and 1.8 g/L, making the water conditions mainly brackish. Combined with the data analysis from this study, it can be seen that the TDS of the lake water was further reduced, and the role of the ecological supplement water was more pronounced. The reason was that the ecological water supply from the Yellow River had a TDS concentration of only 0.676 g/L. Therefore, with the increase in ecological water recharge, the TDS value of Wuliangsu Lake also gradually decreased. The concentrations of ions in groundwater, channel drainage, and lake water decreased successively. From the seasonal variation analysis, the concentration of each ion in the lake in autumn was lower than that in spring, and the concentrations of K^+^ and Ca^2+^ exhibited the most obvious change. The concentrations of K^+^ and Ca^2+^ decreased by 1.04 and 13.5 mg/L, accounting for 15.9% and 17.4%, respectively. The concentration of Ca^2+^ in channel drainage and groundwater showed an upwards trend. In spring, the concentration of Ca^2+^ in channel drainage water was 136.1 mg/L, while in autumn, the concentration of Ca^2+^ increased to 253.5 mg/L, for an increase of 86.3%. The concentrations of Na^+^ and HCO_3_^−^ ions in groundwater displayed the most obvious change, and the concentrations increased by 100.1 and 212.6 mg/L, respectively. This change may be related to crop irrigation in the study area.

#### 3.3.2. Interannual Variation in Ecological Indicators

The interannual changes in the hydrochemical characteristics of Wuliangsu Lake were analysed, as shown in Figure 1, by collecting hydrochemical data [30] of the study area over the years and combining these data with the results of the sampling survey and analyses conducted in 2021.

**Figure 11:**
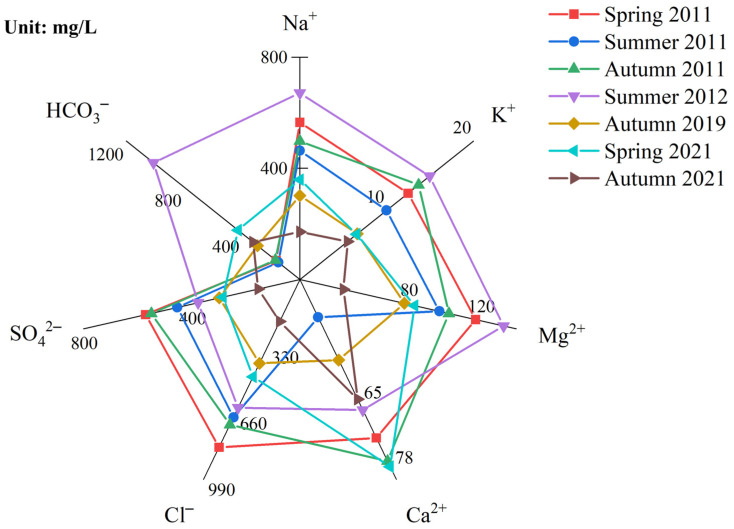
Interannual changes in the main ions of Wuliangsu Lake.

Figure 11 shows the changes in lake water ionic concentrations in recent years, especially between 2011 and 2021. In general, the ion concentration in the lake showed an obvious downwards trend. Among the ions, the concentrations of Na^+^, Cl^−^, and SO_4_^2−^ decreased by 50%, or more than 240 mg/L. Despite an initially low concentration, K^+^ ions also decreased by up to 50%. In addition, HCO_3_^−^ ions showed an upwards trend, increasing by 215.22 mg/L in 2021 compared to 2011, an increase of 57%. There was no significant change in Ca^2+^ ions, and the change of the ionic concentration in the two samples was only 8%. The concentration of various hydrochemical ions in the lake area has decreased significantly since the water from the Yellow River was introduced into the lake as an ecological water supplement, indicating that the ecological water replenishment project has a strong impact on the lake.

**Figure 12:**
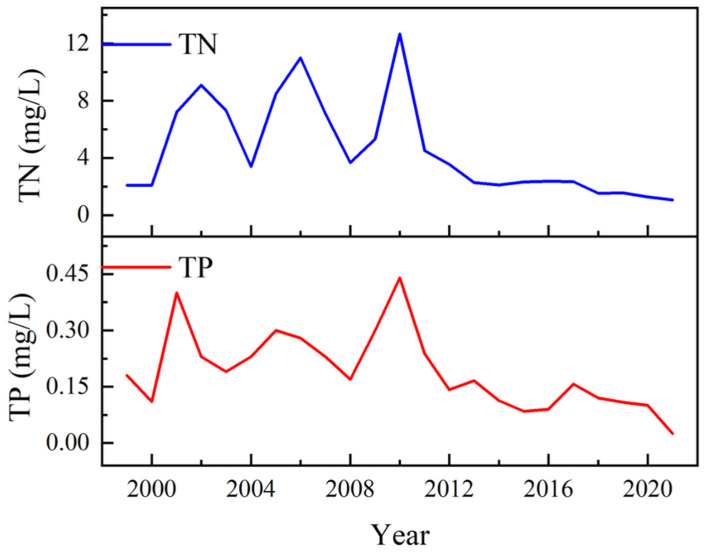
Eutrophication evolution analysis of the long time series of Wuliangsu Lake.

Combined with the eutrophication data of Wuliangsu Lake from 1999 to 2021 [31,32,33], Figure 12 shows the changes in total nitrogen and total phosphorus concentrations in the lake over time by averaging the data of different sampling points in the study area. As shown in the figure, before the start of ecological water supplementation in 2013, the total nitrogen concentration in the lake showed obvious fluctuations. For example, during the period of 1999–2012, the peak concentration was 12.67 mg/L, and the lowest concentration was 2.1 mg/L, which is approximately six times less. However, after 2013, the total nitrogen concentration in the lake significantly decreased, and its volatility was significantly reduced. From 2013 to 2021, the concentration of total phosphorus in the lake was similar to that of total nitrogen, showing periodic fluctuations before 2013, with a maximum difference of 0.3 mg/L between the highest concentration and the lowest concentration. After the implementation of ecological water supplements in 2013, the total phosphorus concentration showed a significant downwards trend, varying between 0.03 and 0.17 mg/L. Ecological water replenishment has achieved significant effects on the improvement of the lake ecological environment.

## 4. Conclusions

The equilibrium model of water and salt was developed by analyzing the equilibrium factors of natural elements and human activities. Additionally, based on the stable isotope information of δ^18^O and δD, the direct interaction between groundwater and surface water was explored by using the MixSIAR model based on Bayesian theory. The results of the study show that the two methods were consistent in quantitative and qualitative analysis. In particular, channel drainage was the main water supply source, followed by atmospheric precipitation, which remained stable at 15% to 20% on average for many years, while the contribution of groundwater to lake recharge was less than 5%. The annual salt content of the groundwater that enters Wuliangsu Lake is 5.9 × 10^4^ t, which accounts for 4.47% of the total salt recharge. Since ecological water replenishment began, the TDS value of the lake water decreased from 1.7 to 1.28 g/L, and the concentrations of total nitrogen and total phosphorus decreased by 71.7% and 58.6%, respectively. In terms of water quality and quantity, groundwater had little influence on lake water, and the ecological water replenishment played a crucial role in the improvement of lake water ecology, which must be considered when allocating water resources for ecological and environment management.

## Figures and Tables

**Table 1:** Water balance analysis for Wuliangsu Lake.

Water Recharge Types	Water Recharge Amount (Unit: 10^8^ m³)	Water Discharge Types	Water Discharge Amount (Unit: 10^8^ m³)
Channel drainage	5.49	Channel drainage	1.86
Precipitation	0.58	Evapotranspiration	3.71
Groundwater	0.25	Groundwater seepage	0.66
Total	6.32	Total	6.23

## Data Availability

Not applicable.

## Supplementary Materials

The following supporting information can be downloaded at https://www.mdpi.com/article/10.3390/ijerph191912202/s1: Table S1. δD-δ^18^O sampling data of different water bodies in April 2021; Table S2. δD-δ^18^O sampling data of different water bodies in October 2021; Table S3. Contribution rate of different water bodies to lake recharge based on MixSIAR (sampled in April 2021); Table S4. Contribution rate of different water bodies to lake recharge based on MixSIAR (sampled in October 2021); Table S5. Data of ion concentration of different water bodies in April 2021 (Unit: mg/L); Table S6. Data of ion concentration of different water bodies in October 2021 (Unit: mg/L).
