# Supplementary material for: Analysis of the Interaction between Lake and Groundwater Based on Water–Salt Balance Method and Stable Isotopic Characteristics
Source: Int J Environ Res Public Health. 2022 Sep 26;19(19):12202. doi: 10.3390/ijerph191912202 (PMC9564894; doi:10.3390/ijerph191912202)
Supplement: Supplementary file 1 [file ijerph-19-12202-s001.zip › ijerph-1906530-supplementary.pdf]

Analysis of the interaction between lake and groundwater based on water balance method and stable isotopic characteristics

Changming Cao [1], Na Li [2], Weifeng Yue [1,*], Lijun Wu [1], Xinyi Cao [1], Yuanzheng Zhai [1]

1 College of Water Sciences, Beijing Normal University, Beijing 100875, China; 202121470002@mail.bnu.edu.cn (C.C.); yuewf@bnu.edu.cn (W.Y.); 202021470026@mail.bnu.edu.cn (L.W.); 202021470003@mail.bnu.edu.cn (X.C.); zyz@bnu.edu.cn (Y.Z.)
2 China Irrigation and Drainage Development Center, Beijing 100054, China; gpzxlina@126.com
* Correspondence: yuewf@bnu.edu.cn

Supplementary Materials

Table S1. δD-δ^{18}O sampling data of different water bodies in April 2021.

Table S2. δD-δ^{18}O sampling data of different water bodies in October 2021.

Table S3. Contribution rate of different water bodies to lake recharge based on MixSIAR (sampled in April 2021).

Table S4. Contribution rate of different water bodies to lake recharge based on MixSIAR (sampled in October 2021).

Table S5. Data of cion concentration of different water bodies in April 2021 (Unit： mg/L).

Table S6. Data of cion concentration of different water bodies in October 2021 (Unit： mg/L).

Table S1. δD-δ^{18}O sampling data of different water bodies in April 2021.

		Max (‰)	Min (‰)	Mean (‰)	CV
Channel drainage	δD	-56.17	-74.14	-64.38	-11.31
	δ^{18}O	-6.92	-10.24	-8.28	-17.22
Groundwater	δD	-55.90	-78.84	-69.23	-10.18
	δ^{18}O	-7.13	-10.57	-9.26	-10.97
Lake water	δD	-53.59	-62.89	-58.80	-5.19
	δ^{18}O	-6.47	-8.21	-7.46	-7.76

Table S2. δD-$\delta^{18}O$ sampling data of different water bodies in October 2021.

		Max (‰)	Min (‰)	Mean (‰)	CV
Channel drainage	δD	-51.71	-70.93	-65.51	-22.15
	$\delta^{18}O$	-4.59	-10.15	-8.74	-11.38
Groundwater	δD	-43.66	-80.41	-67.70	-15.76
	$\delta^{18}O$	-6.96	-11.00	-9.32	-13.68
Lake water	δD	-51.03	-69.76	-64.83	-9.55
	$\delta^{18}O$	-5.80	-9.89	-8.80	-15.30

Table S3. Contribution rate of different water bodies to lake recharge based on MixSIAR (sampled in April 2021).

	Mean	SD	2.50%	5%	25%	50%	75%	95%	97.50%
Epsilon.1	0.591	0.445	0.154	0.182	0.319	0.479	0.729	1.365	1.673
Epsilon.2	0.619	0.432	0.161	0.191	0.338	0.512	0.768	1.442	1.732
Groundwater	0.079	0.065	0.003	0.006	0.029	0.049	0.113	0.205	0.236
Precipitation	0.21	0.07	0.073	0.095	0.167	0.210	0.251	0.322	0.349
Drainage channel	0.711	0.086	0.534	0.565	0.659	0.741	0.768	0.841	0.871

Table S4. Contribution rate of different water bodies to lake recharge based on MixSIAR (sampled in October 2021).

	Mean	SD	2.50%	5%	25%	50%	75%	95%	97.50%
Epsilon.1	0.444	0.347	0.082	0.109	0.218	0.353	0.555	1.094	1.411
Epsilon.2	0.602	0.449	0.122	0.149	0.303	0.485	0.762	1.448	1.839
Groundwater	0.059	0.084	0.002	0.004	0.02	0.042	0.072	0.144	0.183
Precipitation	0.164	0.081	0.016	0.031	0.108	0.181	0.217	0.303	0.328
Drainage channel	0.777	0.115	0.57	0.626	0.729	0.777	0.843	0.922	0.948

Table S5. Data of cion concentration of different water bodies in April 2021 (Unit: mg/L).

Indicators		Ca^{2+}	Mg^{2+}	Na^{+}	K^{+}	HCO_3^-	SO_4^{2-}	Cl^-	NO_3^-
Lake	Max	94.20	113.31	566.69	7.92	523.96	357.76	818.63	6.15
	Min	68.13	68.05	265.24	5.17	194.94	228.61	313.82	0.51
	AVG	77.36	83.50	359.20	6.53	427.56	283.88	485.90	1.53
	SD	6.71	14.92	93.41	0.84	87.90	39.20	143.58	1.51
Ground water	Max	396.53	314.67	5488.12	35.30	3038.78	1981.54	6713.88	189.45
	Min	22.31	38.83	120.80	2.92	320.97	180.81	148.49	0.30
	AVG	187.78	175.23	1432.37	12.89	1288.95	955.49	1617.04	47.76
	SD	124.17	102.64	1520.65	9.25	867.14	564.09	1841.14	65.66
Channel drainage	Max	417.79	451.12	4490.63	13.73	1664.77	1461.17	6970.13	20.75
	Min	58.83	31.97	78.24	2.39	281.51	115.36	72.61	0.76
	AVG	136.12	168.38	1036.16	6.35	766.08	519.08	1515.02	5.83
	SD	106.84	134.69	1308.28	3.37	508.28	413.12	2040.08	5.81

Table S6. Data of cion concentration of different water bodies in October 2021 (Unit: mg/L).

Indicators		Ca^{2+}	Mg^{2+}	Na^{+}	K^{+}	HCO_3^-	SO_4^{2-}	Cl^-	NO_3^-
Lake	Max	75.17	100.61	388.82	8.10	590.08	268.86	475.00	6.74
	Min	40.88	28.48	91.59	3.86	139.35	123.37	117.07	0.09
	AVG	63.91	44.07	171.83	5.49	323.75	153.83	209.74	3.65
	SD	11.18	23.16	89.30	1.26	117.43	47.26	109.86	2.37
Ground water	Max	542.88	376.13	6163.68	78.22	2578.00	2106.40	8161.04	224.22
	Min	43.02	60.62	131.43	3.60	646.74	178.23	144.22	1.76
	AVG	183.23	185.65	1532.45	23.16	1501.57	979.59	1613.48	85.56
	SD	156.31	118.10	1693.56	22.48	609.10	617.64	2296.15	77.38
Channel drainage	Max	1515.29	1131.17	5513.92	23.20	2972.60	1543.62	11689.4	11.88
	Min	67.22	33.81	75.40	2.88	300.68	125.45	67.31	0.20

AVG	253.53	202.15	898.09	7.33	870.00	418.89	1620.78	7.00
SD	447.46	334.55	1662.82	5.96	793.26	464.78	3584.72	2.97
